# Ultra-Wideband Compact Fractal Antenna for WiMAX, WLAN, C and X Band Applications

**DOI:** 10.3390/s23094254

**Published:** 2023-04-25

**Authors:** Mohamed Marzouk, Youssef Rhazi, Ibrahime Hassan Nejdi, Fatima-Ezzahra Zerrad, Mohamed Saih, Sarosh Ahmad, Adnan Ghaffar, Mousa Hussein

**Affiliations:** 1 Microelectronics, Embedded Systems and Telecommunications (MiSET), Faculty of Sciences and Technology, Beni-Mellal 23000, Morocco; mohamed.marzouk@usms.ma (M.M.); y.rhazi@usms.ma (Y.R.);; 2 Automatic and Energy Conversion (AEC), Faculty of Science and Technology, Beni-Mellal 23000, Morocco; ibrahime.nejdi@usms.ma; 3 Laboratory IMII, Faculty of Sciences and Techniques, Hassan First University of Settat, Settat 26000, Morocco; f.zerrad@uhp.ac.ma; 4 Department of Electrical Engineering and Technology, Government College University Faisalabad, Faisalabad 38000, Pakistan; saroshahmad@ieee.org; 5 Department of Signal Theory and Communications, Universidad Carlos III de Madrid (UC3M), 28911 Madrid, Spain; 6 Department of Electrical and Electronic Engineering, Auckland University of Technology, Auckland 1010, New Zealand; aghaffar@aut.ac.nz; 7 Department of Electrical Engineering, United Arab Emirates University, Al Ain 15551, United Arab Emirates

**Keywords:** ring antenna, ultra-wideband, wireless communications, transmission-line, Ansys HFSS simulation, slot antenna, microstrip patch antenna

## Abstract

In this paper, a compact dual-wideband fractal antenna is created for Bluetooth, WiMAX, WLAN, C, and X band applications. The proposed antenna consists of a circularly shaped resonator that contains square slots and a ground plane where a gap line is incorporated to increase the gain and bandwidth with a small volume of 40 × 34 × 1.6 mm^3^. The patch was supported by the FR4 dielectric, which had a permittivity of 4.4 and tan δ = 0.02. A 50 Ω microstrip line fed this antenna. The antenna was designed by the HFSS program, and after that, the simulated results were validated using the measured results. The measurement results confirm that the suggested antenna achieves dual-band frequencies ranging from 2.30 to 4.10 GHz, and from 6.10 GHz to 10.0 GHz, resonating at 2.8, 3.51, 6.53, and 9.37 GHz, respectively, for various applications including commercial, scholarly, and medical applications. Moreover, the antenna’s ability to operate within the frequency range of 3.1–10.6 GHz is in accordance with the FCC guidelines for the use of UWB antennas in breast cancer detection. Over the operational bands, the gain varied between 2 and 9 dB, and an efficiency of 92% was attained. A good agreement between the simulation and the measured results was found.

## 1. Introduction

Globally, wireless communications are expanding rapidly. Given the speed of installation of wireless networks compared to that of building wired infrastructure to cover a specific area, wireless equipment is becoming more affordable and simpler to use [1,2]. Microstrip antennas are the most widely used antennas to provide ultra-wideband operation. They have attracted significant attention from companies and universities in recent years due to their advantages such as low transmission power, high bandwidth, omnidirectional pattern, high data rate, and low price [3,4].

In response to the need for compact and high-performance antennas, antenna designers have developed a new type of patch antenna that incorporates fractal geometries. Fractal geometries are composed of numerous segments that are precisely alike but with varying size scales. This concept was first introduced in 1983, and antenna designers are now utilizing it to create antennas that outperform traditional patch antennas. By combining fractal shapes with patch antennas, a multiband frequency antenna with high gain can be achieved. The incorporation of capacitive and inductive loads onto the patch surface enhances the antenna’s frequency operation, resulting in a wider bandwidth and a multiband response.

The need for wider bandwidth, multiband, and low-profile antennas is rapidly increasing, with both the commercial and military fields requiring multifunctional communication systems that can operate at multiple frequency bands.

The literature describes many strategies for creating multiband operations to accommodate developmental requirements [5,6,7,8,9,10,11,12,13,14,15,16,17,18,19,20,21,22,23,24,25,26,27,28,29,30,31,32,33,34]. In order to attain a larger working bandwidth and a smaller dimension, fractal geometry is applied in ref. [5]. Numerous fractal antenna methods exist for multiband operation, such as those of Minkowski [6], Koch Curves [7], Mandelbrot [8], Sierpinski [9], Hilbert [10], the use of fractal tree antennas [11], the method of Cantor [12], and the use of shaped fractals [13,14,15]. As opposed to a regular antenna, which can only operate in one band, a fractal antenna can operate in several bands. Therefore, fractal antennas have many applications. With slotted antennas, good performance can be attained [16,17,18].

Fractals are recursive in nature [19,20]. The self-similarity characteristic of fractal geometries facilitates the attainment of broadband and multiband properties. The characteristics of the fractal patch allow a change in the direction of the current flow and an extension of it over a longer distance, which results in the creation of new resonant frequencies and subsequently a UWB operation with radiation patterns and input impedance comparable to those of conventional antennas of a larger size. The authors of [21] created a fractal antenna based on the Cantor sequence that operates in the ISM band 2.40 GHz. The antenna is a suitable candidate for the medical field. M. Madi and other authors in [22] used a dual-band fractal antenna with a bandwidth of about 500 MHz for breast cancer detection applications.

A number of applications, including Wi-Fi, biomedical applications, C band, radar, satellite communication, and wireless computer networks, are covered by the work mentioned by Anita Garhwal and others [23]. In order to present an antenna with performance suitable for WLAN and WiMAX applications, the authors of [24] designed and manufactured a circular-slot antenna which functions over two frequency bands, 2.8–3.9 GHz and 5.2–6.2 GHz. Ref. [25] presents a miniature quad-band antenna that has a gain of 8.12 dB with 57 × 31 × 1.6 mm^3^ reduced dimensions.

The researchers of [26] used a 4 × 4 MIMO antenna with four components placed on either side of the dielectric material to operate in the 2.40–2.48 GHz and 3.40–3.60 GHz bands. For dual-band wireless applications, Som Pal Gangwar et al. [27] describe the design of a slot-modified antenna. By using three different types of slots, [28] shows a small switchable hexagonal-shaped monopole antenna for multiband and UWB operation with the dimensions 36 × 36 × 1.6 mm^3^. The reconfigurable antenna is employed by the researchers of [29,30,31], and these structures provide multifrequency operations with good performances. A hexagonal-ring fractal antenna was created by the authors of [32] to operate in four distinct frequency bands, with resonant frequencies of 1.7, 2.4, 3.1, 4.5, and 6 GHz. The antenna displays favorable radiation properties and reasonable gain, indicating that it is a viable option for wireless applications. The article in [33] analyzes a new compact and low-cost antenna designed for specific microwave applications, such as WLAN 802.11 a/b/g and WiMAX applications, using a modified ground structure to enhance its multiband performance. Shuo Liu and others introduced in [34] a novel design for a dual-layer, dual-band patch antenna with linear polarization that utilizes E-shaped and U-slot patches to achieve dual-band performance. Specifically, the antenna was designed to operate within the WLAN (2.40–2.48 GHz) and WiMax (3.40–3.61 GHz) frequency bands.

There are several methods for enhancing antenna performance and increasing bandwidth, including implementing aperture coupled feeding or other feeding techniques to improve impedance matching and increase bandwidth [35], incorporating frequency-selective surfaces (FSS) or electromagnetic-bandgap (EBG) structures can suppress surface waves and increase antenna bandwidth [36], employing multiple-input multiple-output (MIMO) technology, which uses multiple antennas to increase the system’s capacity and enhance performance [37], and adding parasitic elements, such as directors or reflectors, to the antenna structure to increase gain and bandwidth [38]. These methods can be used alone or in combination to achieve the desired performance and bandwidth characteristics for a given application.

This research exhibits the design and fabrication of a fractal antenna with a broadband characteristic by combining fractal slots and a partial ground plane. FR-4 is used as a support for the proposed antenna due to its high electrical insulation properties that prevent the flow of electric current between different parts of the antenna. Moreover, FR-4 is a cost-effective and readily available substrate, which makes it a popular choice for antenna fabrication. With an overall size of 40 × 34 × 1.6 mm^3^, the proposed antenna is a novel solution for various applications due to its unique design and capabilities. The antenna achieves dual-band frequencies from 2.30 to 4.10 GHz and from 6.10 GHz to 10.0 GHz, offering wideband capability that provides greater flexibility and versatility. Additionally, the antenna’s compact size results in improved efficiency and reduced costs, making it a potential solution for portable devices when space is limited. In wireless communications, the antenna’s dual-band frequencies make it suitable for WiMAX and WLAN applications, allowing faster data transfer rates and wider coverage areas. For military communication systems, the antenna’s wideband capability and compact size make it a reliable and flexible solution across different frequencies and platforms. The antenna’s dual-band frequencies and wideband capability also make it a potential option for reliable and efficient communication in radar systems across different frequencies and ranges. Overall, the proposed antenna represents a significant advancement in antenna design, offering unique benefits and potential applications. The planned antenna radiates bidirectionally in the E- and H-planes with a high gain that reaches 9 dB and excellent efficiency of up to 92%.

Section 2 describes the antenna’s geometry and the process of the design’s evolution. A parametric study of the suggested antenna is presented in Section 3. In Section 4, an explanation of the simulation and experiment results is developed in detail. The final section of this research contains the conclusion.

## 2. Antenna Design

### 2.1. Antenna Dimensions

**Figure 1:**
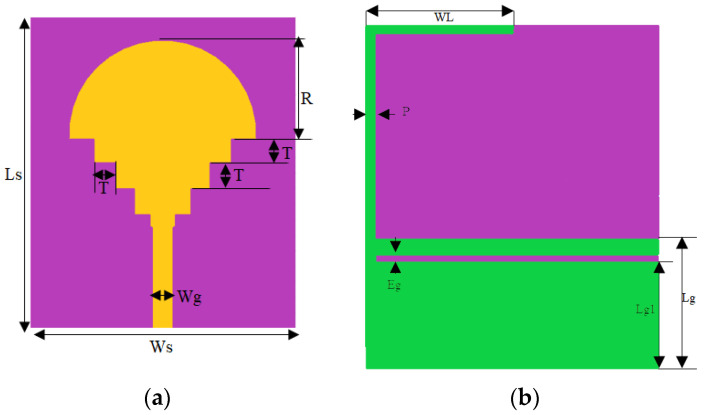
(**a**) The front plane, and (**b**) the ground plane of the suggested antenna.

The suggested fractal antenna was produced by going through several iterations, using a circularly shaped patch as an initiator, and in each step square-shaped slots were introduced as shown in Figure 1. The FR4 dielectric was used as a support for the suggested antenna. The primary goal of designing the circular antenna was to make it resonate at a frequency of 3.6 GHz to provide coverage for both WiMAX and WIFI. To determine the antenna’s size, Equations (1) and (2) outlined in reference [39] were utilized. Once the antenna dimensions were optimized to cover the desired frequency range, a radius of 12 mm was identified as the optimal size. The power was provided by a 50 Ω line where the size was calculated by the transmission line model equation. The ground plane was partial with a gap line. The antenna was of a width (Ws) of 40 mm and a length (Ls) of 34 mm. Table 1 summarizes all suggested antenna sizes.

(1) $$R=\frac{F}{\sqrt{1+\left(\frac{2h}{{\pi \varepsilon }_{r}F}\right)\left[\ln\left(\frac{\pi F}{2h}\right)+1.7726\right]}}$$

where:

(2) $$F=\frac{8.791\times 10^{9}}{f_{r}\sqrt{\varepsilon_{r}}}$$

### 2.2. Design Evolution Methodology

**Figure 2:**
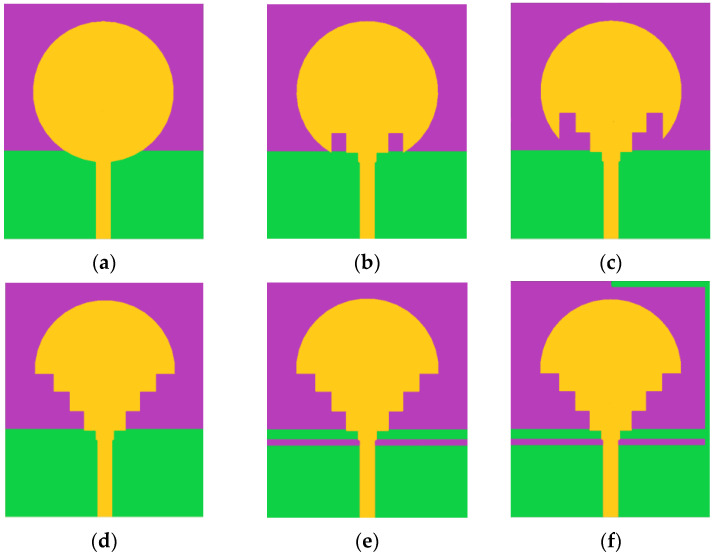
Steps taken to achieve the ultimate design of the suggested antenna.

The evolution of the fractal patch design is shown in Figure 2a–f. The initiator was presented by the fundamental circular antenna with a partial ground with resonant frequencies at 5.87 GHz and 6.35 GHz.

The second step was to remove four squares with 3.33 mm edges from the feed line side, to disturb the current distribution of the antenna, create new resonant frequencies and generate a wider bandwidth (of 2.15 GHz). The size of the squares was determined to be 3.33 mm, based on simulation results and optimization processes aimed at achieving the desired performance parameters for the antenna design. Four more rectangular sections were cut from the patch in the same size as that cut in the third iteration. The choice of four squares was determined through a trial-and-error approach during the iterative procedure, where various configurations were tested to improve the antenna’s performance. This iteration offers four resonance frequencies of 0.29 [1.84, 2.13] GHz, 1.06 [4.49, 5.55] GHz, 0.39 [5.98, 6.37] GHz, and 2.09 [8.95, 11.04] GHz

**Figure 3:**
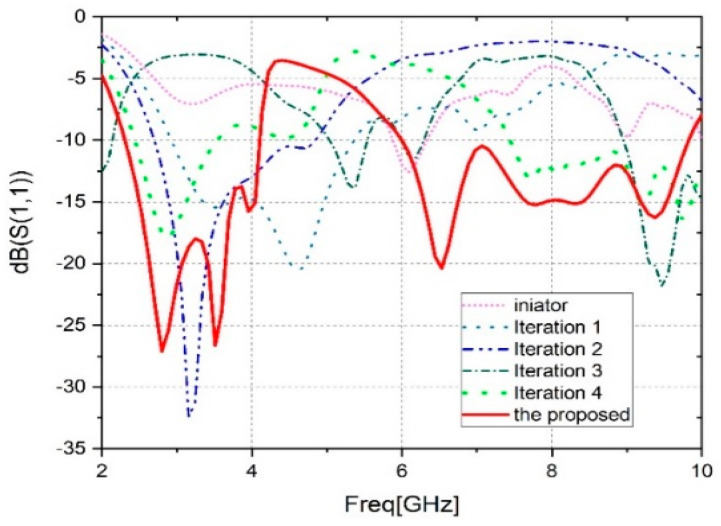
Comparison of the S11 at different design stages.

In the fourth iteration, the ground was modified by a rectangular slit with a 1 mm thickness, and this modification offers two operating bands, [2.43, 3.54] GHz and [7.45, 10.79] GHz. In the proposed antenna, a ‘‘L” shape is welded on the partial ground that gives two wide bands with impedances of 1.84 [2.26, 4.10] GHz and 3.9 [6.10, 10] GHz, as shown in Figure 3.

This patch is applicable to the WLAN, C, and X bands, Bluetooth, and WiMAX. To clarify, the proposed antenna was designed to operate within the frequency ranges typically associated with C and X bands. While the frequency range of 2.30 GHz to 4.10 GHz technically falls under the S-Band, it is sometimes referred to as the lower C-Band (3.7–3.98 GHz). Additionally, the frequency range of 6.10 GHz to 10.0 GHz partially falls within the IEEE-defined C-Band range and is also considered part of the X-Band. Therefore, while the antenna may not cover the entire spectrum of C and X bands, it is compatible with many applications within these frequency ranges. It is concluded that during the antenna’s evolution, the S-parameter curves are improved. The antenna evolution results are presented in Table 2.

The suggested antenna has was the subject of comprehensive study to optimize its design and it was found that its current design offers the best bandwidth performance. Through a parametric study, multiple design options were considered and their effectiveness was tested to arrive at this conclusion. Therefore, the current design appears to be the most efficient and effective option for achieving the desired bandwidth.

### 2.3. Analysis of the Suggested Antenna Parametric

The proposed fractal antenna has a wideband operation and improved gain. These parameters were reached through a parametric study using HFSS electromagnetic solver high-frequency structure simulator. By changing just one parameter while holding the other parameters constant, the suggested antenna performance was tested.

A. Effect of the rayon, R

**Figure 4:**
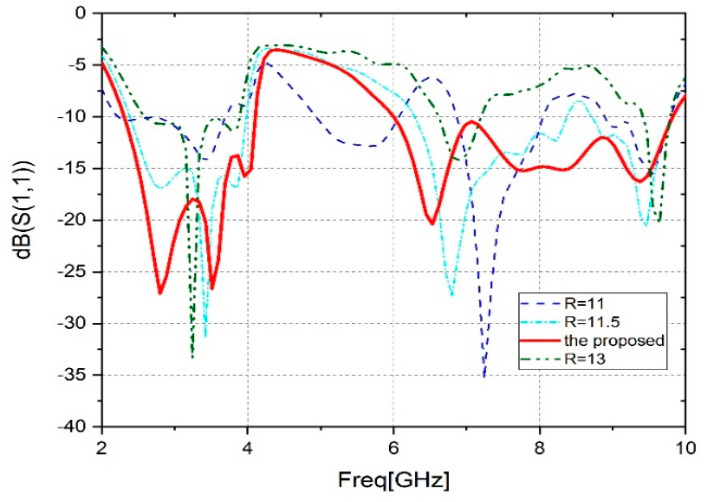
Parametric analysis of radius (R).

The antenna patch radius is crucial in determining how much the resonant frequencies vary. Figure 4 shows the suggested structure’s impact on the reflection coefficient using three different rayons. The R is modified from 11 mm to 13 mm with a 1 mm step. The impact of the radius on the antenna performance is clearly observed by analyzing Figure 4. There is a reduction in the frequency range covered by the antenna. Also, a band shift is noted from 4 GHz to 10 GHz. However, a wide band is achieved for R = 12 mm.

B. Effect of the position of Lg1

**Figure 5:**
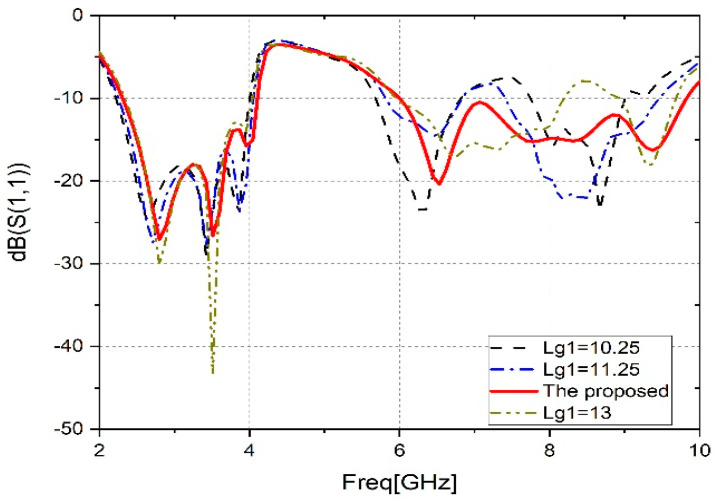
Parametric analysis (Lg1).

To further improve the performance of the antenna, a slit in the ground plane was inserted. The change in slot position in the partial ground has a significant effect on the wideband operation of the monopole antenna. A decrease in bandwidth is observed when shifting from the suggested value of ‘Lg1’, as shown in Figure 5. It is clear that Lg1 = 12.25 mm is the best.

C. Effect of the position Lg1

**Figure 6:**
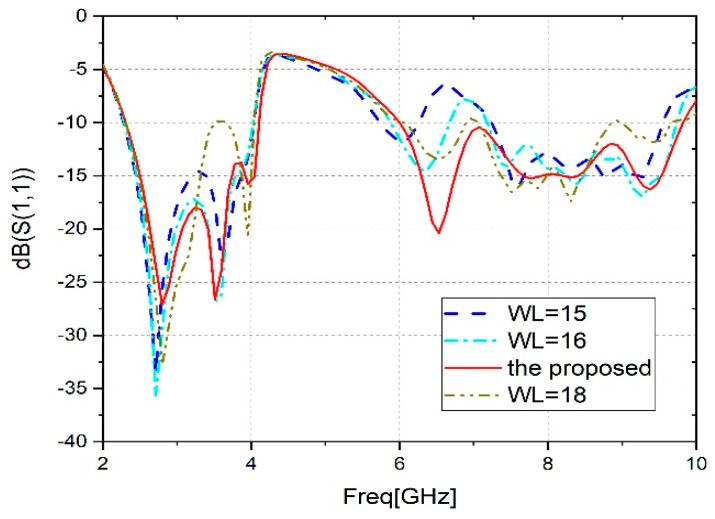
Parametric analysis (WL).

Figure 6 shows the parametric analysis of WL. It is noticeable that the modification of ‘WL’ has no effect on the first frequency band ([2.30, 4.10] GHz). While it has a major effect on the second band, it moves away from the proposed value. In conclusion, to provide wideband operation, excellent impedance, low prices, and compact size to cover the latest wireless communication systems, the proposed value of WL = 17 mm is the best choice.

**Figure 7:**
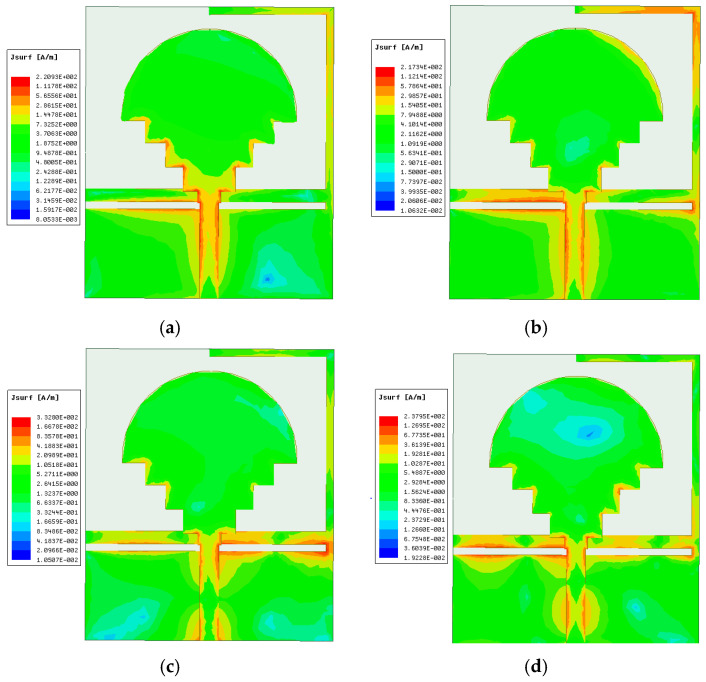
Surface current distribution at (**a**) 2.8 GHz, (**b**) 3.51 GHz, (**c**) 6.53 GHz, and (**d**) 9.37 GHz.

Figure 7 illustrates the surface current distribution of the suggested antenna at 2.8 GHz, 3.51 GHz, 6.53 GHz, and 9.37 GHz simulated by HFSS. The surface current distribution of a fractal antenna can be characterized by its multifractal properties, where the current density varies at different points on the antenna surface. The self-similar nature of fractal antennas results in a uniform distribution of the surface current over the antenna structure, which helps to reduce the inhomogeneity of the electromagnetic field. Moreover, the fractal nature of these antennas allows the creation of multiple resonant frequencies, which makes them suitable for multi-band applications. The surface current distribution of a fractal antenna is critical in determining its radiation properties, such as directionality and polarization, and can be optimized for specific performance requirements. This analysis is performed to understand the wideband operation of the patch. In Figure 7a,b,d, it is observed that the current distribution is mostly concentrated at the slot of the partial ground, feed line, and the lower edge of the patch. This is because the slots create additional paths for current flow and enhance the current density in those regions. At a frequency of 3.51 GHz, the current is also distributed at the ‘L’ shape of the ground, which causes the wideband properties of the antenna. The ‘L’ shape creates an additional resonator that adds to the existing ones, thereby increasing the bandwidth of the antenna. When the slots are integrated into the antenna, the main current flow splits and creates new paths. This causes the antenna to become electrically larger in size and creates different resonators, which subsequently leads to the wideband operation of the antenna. The wideband operation is due to the presence of multiple resonant modes that contribute to the radiation of the antenna over a wide range of frequencies.

## 3. Results and Discussion

**Figure 8:**
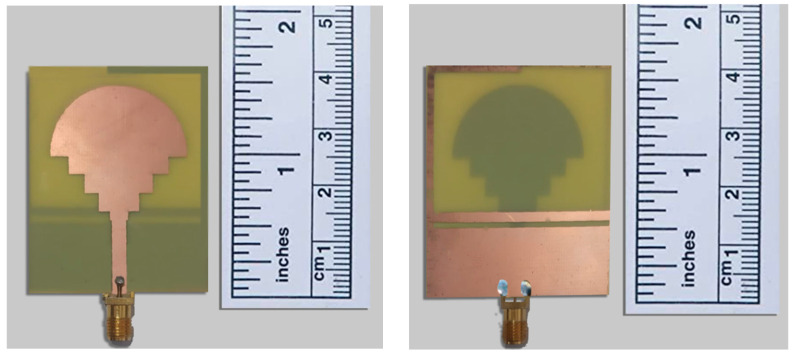
Front and rear face of the fabricated antenna.

The antenna was designed and optimized using the HFSS software 17.2, which is based on the finite element approach (FEM). An FR4 substrate of the optimal size of 40 × 34 mm^2^ was used to fabricate the suggested fractal antenna to validate the simulation results. Figure 8 shows the top and bottom views of a fabricated antenna.

**Figure 9:**
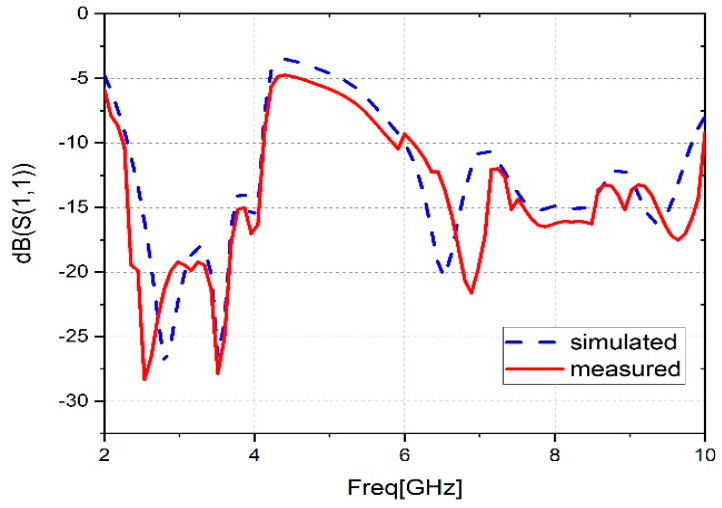
Comparison of simulated and measured S11.

Using a vector network analyzer, the suggested antenna reflection coefficient was measured. Figure 9 displays the comparison between the simulated and measured reflection coefficients of the suggested broadband antenna. The comparison of these results shows that there is good agreement and confirms the wideband operation property. A slight difference is noticed in the higher frequencies; this may be due to the circumstances of manufacturing and measurement. In the Figure 9, a correspondence can be found between the simulated and measured results in the first band, [2.3–4.10] GHz. On the other hand, in the second band, a slight improvement can be observed in the measured results in the high frequencies.

The proposed prototype antenna covers the range of frequencies from 2.26 to 4.10 GHz and from 6.10 to 10 GHz with an impedance bandwidth of 1.84 and 3.90 GHz successively, and with reflection coefficients of −28.38 dB, −27.62 dB, −21.94 dB, and −17.45 dB, respectively. The suggested antenna matches the bandwidth requirements of several wireless protocols, such as WiMAX (2.5–2.69, 3.3–3.8, and 5.25–5.825 GHz), WLAN (2.4–2.484 and 5.15–5.825 GHz), C band (6–8 GHz), and X band (8–10 GHz).

**Figure 10:**
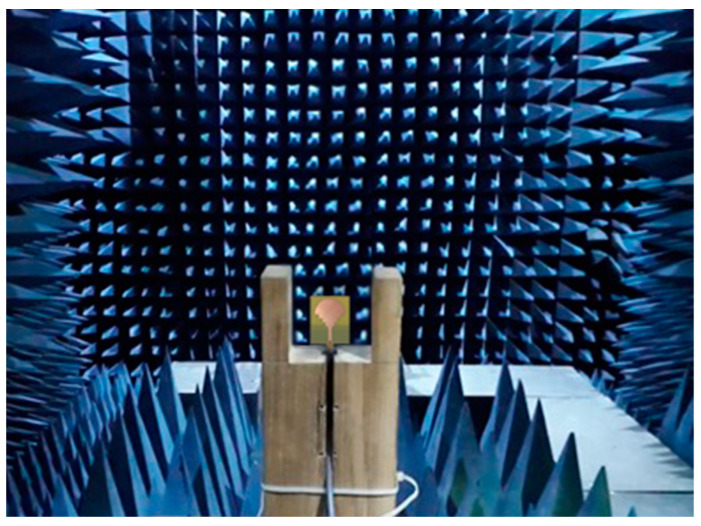
Suggested antenna measurement setup inside the anechoic chamber.

**Figure 11:**
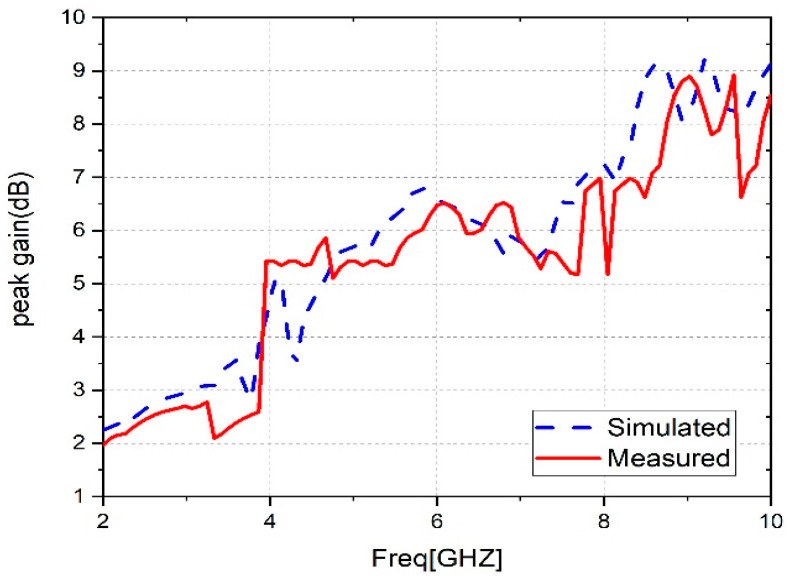
Peak gain measurements and simulations for the suggested antenna.

An anechoic chamber, such as that seen in Figure 10, was used to measure the maximum gain, efficiency, and radiation pattern of the proposed antenna. The peak gain of an antenna depends on several factors, including its size, shape, and radiation pattern. It is an important parameter in the design of antennas, as it determines the antenna’s ability to transmit or receive signals in a specific direction. The higher the peak gain of an antenna, the stronger its signal will be in the desired direction, which is particularly important in applications such as satellite communications, radar applications, and wireless networks. Figure 11 displays the comparison between the measured and simulated peak gain results of the suggested antenna for the frequency range of 2 to 10 GHz. The antenna provides impressive results, with a maximum simulated peak gain of 9.2 dB at the frequency of 9.3 GHz, whereas the maximum measured is 9 dB at the frequency of 9.5 GHz.

**Figure 12:**
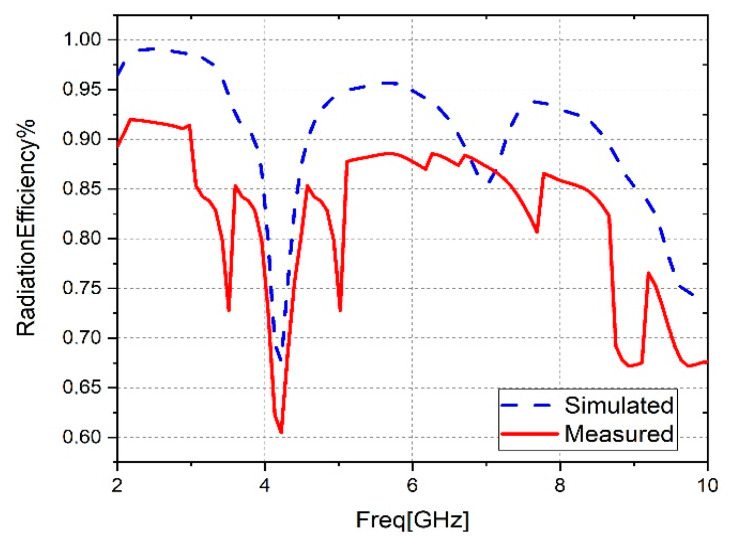
Radiation efficiency measurements and simulations for the suggested antenna.

The comparison between the measured and simulated radiation efficiency of the antenna is presented in Figure 12. Radiation efficiency is a measure of the ability of an antenna to convert the electrical power that is supplied to it into electromagnetic radiation that propagates through free space. It is defined as the ratio of the power radiated by the antenna to the total input power supplied to it. In other words, radiation efficiency measures how effectively an antenna is able to convert the electrical energy it receives into useful radiation while minimizing the energy lost as heat or stored in the antenna structure. The radiation efficiency of an antenna is influenced by several factors, such as the antenna’s size, shape, material properties, and the presence of nearby objects that can reflect or absorb the radiated energy. The proposed antenna can reach a simulated radiation efficiency of 99% in the operating band. In addition, the radiation efficiency measured reached 92% at 2.3 GHz. The modest discrepancy noted between the simulation and measurement results may have been caused by ambient conditions, manufacturing tolerances, losses at the SMA connector, and impurities in the substrate. However, the results clearly demonstrate good agreement between the simulation and measurement results despite these factors. The measured radiation efficiency of the suggested antenna has dips, which could be attributed to simulation assumptions. These assumptions may not reflect the actual conditions under which the antenna is operating, as the simulation may make assumptions about the antenna’s surroundings, or about the behavior of the transmitted and received signals, which may differ from the real-world conditions.

**Figure 13:**
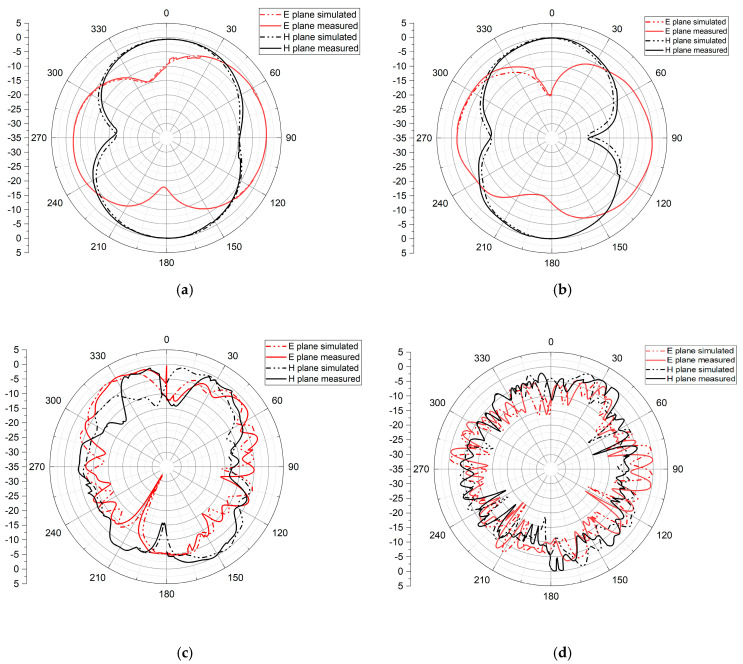
2D radiation pattern at (**a**) 2.8 GHz, (**b**) 3.51 GHz, (**c**) 6.53 GHz, and (**d**) 9.37 GHz.

Figure 13 shows the fractal antenna’s radiation pattern as simulated and measured in the E (ϕ = 0°) and H (ϕ = 90°) planes, measured inside the anechoic chamber and simulated by the HFSS software. The radiation pattern of an antenna describes how electromagnetic radiation is distributed in space when the antenna is transmitting or receiving a signal. It shows the relative strength of the electromagnetic field in different directions from the antenna. Figure 13a–d displays a great deal of agreement between the simulated and measured results at the resonant frequencies of 2.8 GHz, 3.51 GHz, 6.53 GHz, and 9.37 GHz. The radiation patterns of the suggested antenna exhibit different shapes at different frequencies. At lower frequencies, the radiation patterns show bidirectional radiation with an “8” shape in both planes, H and E, indicating the ability to transmit and receive signals in specific directions. As the frequency increases, the radiation pattern becomes quasi-omnidirectional, as demonstrated in Figure 13c,d, meaning that signals are transmitted and received in all directions. These radiation patterns are critical to the practical use of the antenna in various applications. For example, the bidirectional radiation pattern is useful in point-to-point communication systems and radar systems, while the quasi-omnidirectional pattern is more useful in wireless communication applications where signal coverage in all directions is necessary.

Table 3 compares the proposed antenna with other antennas published in the literature in terms of size, substrate type, frequency range, bandwidth, resonance frequency, peak gain, and applications. The mono-bandwidth antenna suggested by [21] has large dimensions of 46.6 × 53.4 mm^2^ but a small bandwidth. The authors of [22] defined a dual-band antenna with a high gain that reaches 10 dB but that is slightly large in size, has a small bandwidth of 0.55 and 0.2 GHz, and its design is complex to manufacture. The antenna provided by [23] is used in wireless computer networks such as Wi-Fi and C-band networks. It has a large size of with dimensions of 60 × 63 mm^2^, with narrow bandwidths and a gain that does not exceed 5.79 dB. The one presented by [25] has a large size with a narrow operational band. Additionally, the patch proposed by [26] has big dimensions and poor bandwidth. In [27], the antenna capable of operating at two different frequencies has considerable dimensions of 48 × 48 mm^2^. However, its bandwidth is limited and does not exceed 250 MHz, and it is suitable for GSM and WiMAX applications. In contrast to the antenna suggested in reference [32], which varies between 1.6–3.8 dB, our designed antenna exhibited a higher gain. According to reference [34], the antenna with the ability to function at two distinct frequencies has large dimensions of 60 × 45 mm^2^. Nonetheless, its bandwidth is constrained and does not surpass 700 MHz, making it appropriate for applications such as WLAN and WiMAX.

Compared to other antennas, the one that is being supplied in this research has great characteristics; it has a compact size, is wideband, and has a high gain, which allows it to cover the operational bands with great efficiency.

## 4. Conclusions

To effectively cover WLAN, C band, X band, Wi-Fi, Bluetooth, and WiMAX applications, this work presents the design and fabrication of an innovative ultra-wideband patch antenna. This antenna has compact dimensions of 40 × 34 × 1.6 mm^3^, and it is supported by an FR-4 substrate to reduce manufacturing costs. The fractal resonator is designed on one side of the substrate. On the other side, a ground plane is implemented. The ground plane is composed of a rectangular part with a slot and a welded ‘‘L’’ shape in order to obtain UWB. After the measurements carried out on the manufactured prototype, dual-wideband operation was achieved; the first has a bandwidth of 1.84 [2.26, 4.10] GHz and the bandwidth of 3.9 GHz [6.10, 10] GHz is for the second band, with four resonant frequencies of 2.8 GHz, 3.51 GHz, 6.53 GHz and 9.37 GHz. In the operational band, this antenna can achieve a radiation efficiency of 92% and a peak gain of 9 dB.

## Figures and Tables

**Table 1:** Ultimate size of fractal antenna proposed.

Parameter	Ws	Ls	Wg	Eg	R	T	P	Lg	Lg1	WL
Values(mm)	34	40	2.6	1	12	3.33	1	15	12.25	17

**Table 2:** Antenna frequency number, loss return, and bandwidth for each iteration.

Iterations	Frequency Number	Loss Return dB	Bandwidth GHZ
The initiator	1	−13.2	0.48 [5.87, 6.35]
Iteration 1	1	−20.43	2.46 [2.9, 5.36]
Iteration 2	1	−32.64	2.15 [2.69, 4.84]
Iteration 3	4	−13.12	0.29 [1.84, 2.13]
		−13.83	1.06 [4.49, 5.55]
		−11.47	0.39 [5.98, 6.37]
		−21.9	2.09 [8.95, 11.04]
Iteration 4	2	−18	1.11 [2.43, 3.54]
		−16.31	3.34 [7.45, 10.79]
The proposed antenna	4	−27.08	1.84 [2.26, 4.10]
		−26.60	
		−20.38	3.9 [6.10, 10]
		−16.28	

**Table 3:** Comparison of the recommended antenna with other antennas described in the literature.

Ref.	Size mm^2^	Substrate	Frequency Range (GHz)	Bandwidth (GHz)	Resonant Frequency (GHz)	Peak Gain (dB)	Methods	Applications
[21]	46.6 × 53.4	FR4	2.42–2.49	0.7	2.45	1.67–1.87	Electromagnetic bandgap structures	Wi-Fi
[22]	50 × 40	FR4	2.24–2.79 3.05–3.25	0.55 0.2	2.45 -	3.75–10	Reducing antenna size and increasing gain through fractal design	Wi-Fi
[23]	60 × 63	FR4	-	0.22 0.71 2.44 1.93	1.62/2.45 4.37/5.56 6.0/8.81	2.18–5.79	Using fractal geometry to achieve multiband operation	Wi-Fi and C band
[24]	40 × 40	FR4	2.88–3.92 5.26–6.28	1.04 1.02	3.38 5.86	0.8–6	Modifying the antenna design to achieve dual-band operation	WiMax and WLAN
[25]	57.2 × 31.2	FR4	0.77–0.83 2.35–2.55 3.05–3.71 4.88–5.81	0.06 0.2 0.65 0.93	0.81 2.45 3.5 5.5	-	Using an asymmetric E-CRLH unit cell design	GSM, WLAN and WiMAX
[26]	60 × 60	FR4	2.4–2.485 3.4–3.6	0.085 0.2	2.46 3.5	-	Incorporating double-sided MIMO	WiMAX and WLAN
[27]	48 × 48	FR4	1.69–1.94 3.64–3.88	0.25 0.24	1.775 3.725	0–7.5	Modifying the antenna design to achieve dual-band operation	GSM and WiMAX
[32]	40 × 32	FR4	1.69–1.88 2.34–2.52 3.07–3.59 4.17–6.26	0.19 0.18 0.52 2.45	1.7 2.4 3.1 4.5 6	1.6–3.8	Incorporating a dumbbell-shaped defected ground structure	GSM, WLAN, and WiMAX
[33]	50 × 50	FR4	2.35–2.44 3.42–3.59 4.82–5.28	0.09 0.17 0.46	2.4 3.5 5.2	-	Using printed circuit board technology	WLAN, and WiMAX
[34]	60 × 45	FR4	2.25–2.95 3.35–3.61	0.7 0.26	2.6, 3.5	-	Using a dual-layer design with an E-shaped and U-slot patch	WLAN and WiMAX
Our Antenna	40 × 34	FR4	2.26–4.10 6.0–9.82	1.84 3.9	2.8 3.51 6.53 9.37	2.1–9	Using fractal geometry, with an ‘L’ shape added to the partial ground plane	Wi-Fi, Bluetooth, WiMAX, WLAN, C and X bands

## Data Availability

Not applicable.
